# IFITM protein regulation and functions: Far beyond the fight against viruses

**DOI:** 10.3389/fimmu.2022.1042368

**Published:** 2022-11-18

**Authors:** Nela Friedlová, Filip Zavadil Kokáš, Ted R. Hupp, Bořivoj Vojtěšek, Marta Nekulová

**Affiliations:** ^1^ Research Centre for Applied Molecular Oncology, Masaryk Memorial Cancer Institute, Brno, Czechia; ^2^ Department of Experimental Biology, Faculty of Science, Masaryk University, Brno, Czechia; ^3^ Institute of Genetics and Molecular Medicine, University of Edinburgh, Edinburgh, United Kingdom

**Keywords:** interferon-induced transmembrane proteins, tumor progression, therapy resistance, stem cells, immunity, tumor immunosurveillance

## Abstract

Interferons (IFNs) are important cytokines that regulate immune responses through the activation of hundreds of genes, including interferon-induced transmembrane proteins (IFITMs). This evolutionarily conserved protein family includes five functionally active homologs in humans. Despite the high sequence homology, IFITMs vary in expression, subcellular localization and function. The initially described adhesive and antiproliferative or pro-oncogenic functions of IFITM proteins were diluted by the discovery of their antiviral properties. The large set of viruses that is inhibited by these proteins is constantly expanding, as are the possible mechanisms of action. In addition to their beneficial antiviral effects, IFITM proteins are often upregulated in a broad spectrum of cancers. IFITM proteins have been linked to most hallmarks of cancer, including tumor cell proliferation, therapeutic resistance, angiogenesis, invasion, and metastasis. Recent studies have described the involvement of IFITM proteins in antitumor immunity. This review summarizes various levels of IFITM protein regulation and the physiological and pathological functions of these proteins, with an emphasis on tumorigenesis and antitumor immunity.

## Introduction

Interferons (IFNs) form a family of cytokines that are released by cells in response to pathogen presence or cell damage/transformation. They act by inducing the expression of hundreds of interferon-stimulated genes (ISGs), including the *IFITM* family encoding well-conserved small transmembrane proteins with a high mutual similarity (1–3). Five functional IFITM homologs have been described in humans – IFITM1, IFITM2, IFITM3, IFITM5 and IFITM10. The immune-related proteins IFITM1, IFITM2 and IFITM3 maintain the highest sequence similarity (4). However, these IFITMs differ in cellular localization and function (5–7). What is the cause of these differences among such similar proteins? Here, we summarize various levels of IFITM regulation, including gene expression, posttranslational modification and oligomerization.

In general, researchers have mainly focused on IFITM proteins and their abilities to block virus infection. This function is probably related to the ability of IFITMs to change membrane properties (8–11). However, the exact mechanism underlying this phenomenon is not yet fully understood. IFITMs also exert other functions worthy of attention, such as their involvement in immune processes and tumor progression which we focus on in this review. IFITM overexpression has been described in various tumor types. Many studies have shown that IFITMs support tumor cell proliferation, migration and invasion (12–17). According to current knowledge, IFITMs are associated with stem cell properties, proper angiogenesis and DNA damage resistance that complicate effective anticancer therapy (18–20). Recent studies have shown a connection between IFITMs and adaptive immunity,particularly their participation in the activation and differentiation of immune cells, as well as changes in their surfaceome (12, 21). How might these functions affect antitumor surveillance? This review summarizes and discusses current knowledge of IFITM proteins, especially the immune-related proteins IFITM1, IFITM2 and IFITM3, different levels of regulation of their expression and function, and their involvement in immune processes and tumor transformation.

## IFITM protein family

Interferons (IFNs) are signaling proteins that are important mediators of innate and adaptive immune responses. Their production is activated by pattern-recognition receptors, which are capable of recognizing molecules frequently expressed by pathogens or molecules released by damaged cells. Secreted IFNs then trigger signaling pathways in autocrine and paracrine manners, leading to the expression of hundreds of genes that are collectively referred to as interferon-stimulated genes (ISGs). These ISGs further affect various cellular processes that generally function to inhibit viral replication (1, 2). However, due to the wide array of responsive genes, IFNs exert pleiotropic biological effects. For example, they affect the proliferation, differentiation and apoptosis of tumor cells and modulate angiogenesis and immune responses. Consequently, they were among the first human proteins shown to be beneficial in cancer therapy (22).

The interferon-induced transmembrane protein (IFITM) family was among the first genes described to be regulated by IFNs. Friedman et al. (23) discovered a new group of proteins induced by IFNs in T98G human neuroblastoma cells by performing differential screening of a cDNA library. This protein group was named the 1-8 protein family, and later alternative names, the IFITM family or Fragilis (which is instead linked to the murine paralogs), were established (24–28).

Lewin et al. (3) isolated three similar genes (*IFITM1*, *IFITM2*, and *IFITM3*) in a single 18-kb genomic DNA fragment and found that these genes are linked at the same locus. Today, the *IFITM* gene family comprises five functional members: *IFITM1* (9-27, Leu-13), *IFITM2* (1-8D), *IFITM3* (1-8U) and *IFITM5* (BRIL, bone-restricted IFITM-like), which are small protein-coding genes clustered within a 26.5-kb region on the short arm of chromosome 11 (11p15.5) in humans. In contrast, the fifth member, *IFITM10*, is also located on chromosome 11 toward the centromere but is not within the cluster containing the other related genes. The murine *Ifitm* family comprises seven functional paralogous genes: *Ifitm1* (alternatively *fragilis2*), *Ifitm2* (*fragilis3*), *Ifitm3* (*fragilis*), *Ifitm5* (*fragilis4*), *Ifitm6* (*fragilis5*), *Ifitm7* and *Ifitm10.* Except for *Ifitm7*, all these genes form the *Ifitm* locus, which spans approximately 67.5 kb on syntenic chromosome 7, while *Ifitm7* is mapped to chromosome 16 (29).

Over the years, IFITM proteins have also been described in species other than humans and mice, e.g., rats, cats and rainbow trout (24–28), and they appear to have an important role in the evolution of vertebrates. IFITM proteins are evolutionarily conserved across vertebrate species from zebrafish to humans, and their rapid evolution was confirmed to be due to selective pressures (4). According to a detailed evolutionary analysis, the vertebrate IFITM family is divided into three subfamilies based on structural and functional characteristics, indicating that these subfamilies evolved from three different ancestors: immune-related IFITMs (including IFITM1, IFITM2 and IFITM3), IFITM5 and IFITM10 (4). The long history of viral infections in vertebrates probably shaped the immune-related IFITM subfamily to its present form. Rapid expansion via tandem duplication occurred during evolution from lower vertebrates to mammals, and positive selection was detected in primates and rodents. This course of evolution refers to important functions of IFITM proteins, providing organisms a selective advantage during evolution (4, 24, 29–32). In general, human IFITM3 appears to exert an inhibitory effect against the widest range of viruses, while IFITM1 and IFITM2 are more specific. Together with data from phylogenetic analyses, IFITM3 appears to be the most ancient antiviral IFITM family member, and progressive evolution has led to duplication and diversification resulting in other immune-related IFITMs (4, 30).

In contrast, IFITM5 and IFITM10 are insensitive to IFNs and exert indistinct antiviral functions (33, 34). IFITM5, which evolved in bony fish, is highly conserved within the IFITM family based on the lack of gene duplication or positive selection detected, consistent with its functional specialization in bone development (4, 31). Little is known about IFITM10 function thus far, but interestingly, all vertebrate IFITM10 genes are divided into two groups: aquatic and terrestrial types. Aquatic vertebrate IFITM10 genes have undergone positive selection and several gene duplications during evolution, implying a possible link to adaptation to aquatic environments (4).

## Gene structure

The sequences of the *IFITM1*, *IFITM2* and *IFITM3* genes are highly homologous, sharing more than 90% identity over 70% of the coding sequence. *IFITM2* and *IFITM3* are very similar (91%), even in noncoding regions (excluding a 68-bp tandem duplication located in the 3' flanking region of *IFITM2*). In contrast, the similarity in the noncoding region between *IFITM1* and the other two genes is only 65%. All three sequences comprise two exons with a single intron located at the same position (3).

**Figure 1:**
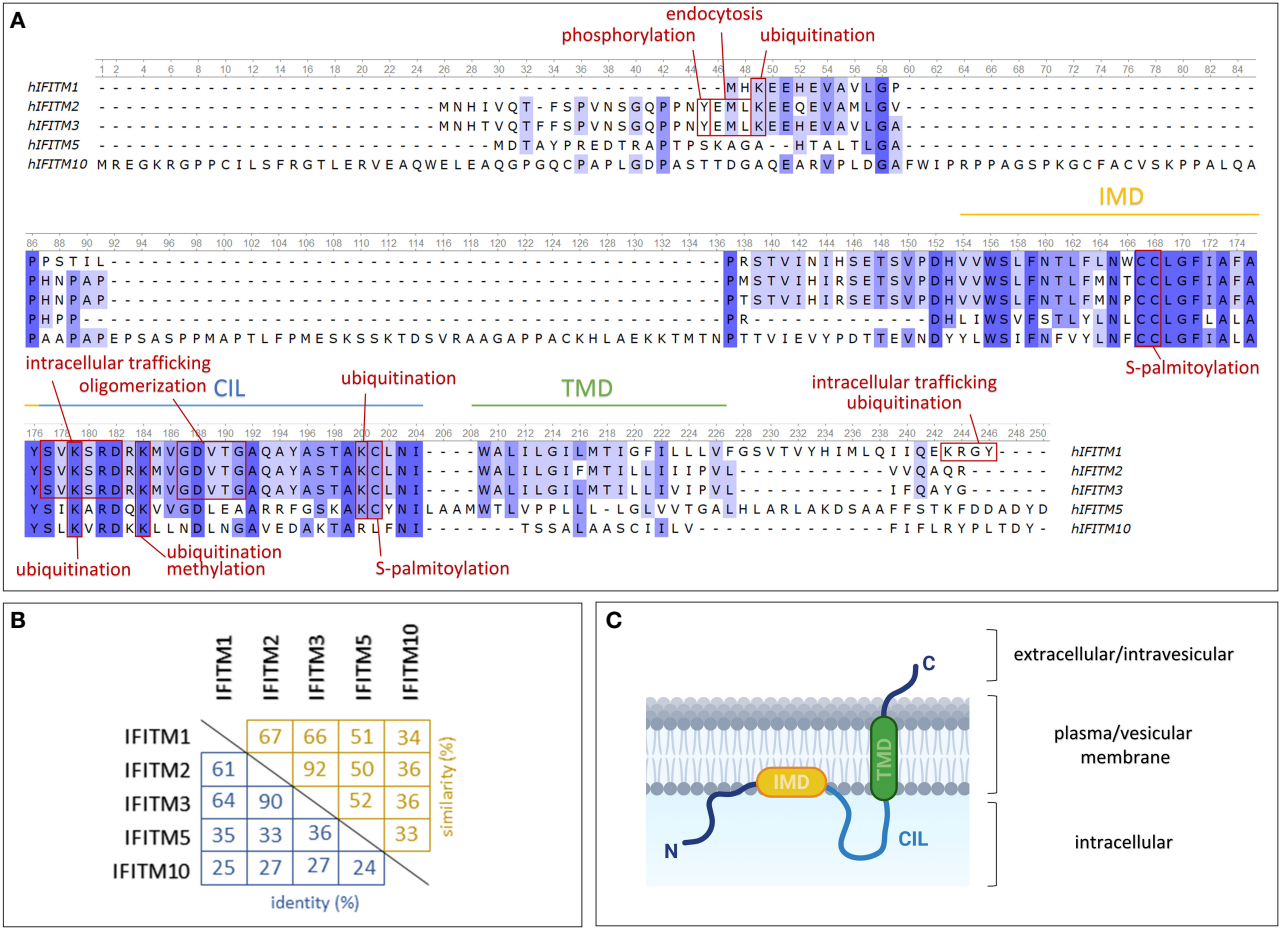
Structures of IFITM protein family members expressed in humans. **(A)** Comparison of protein sequences using a multiple alignment tool (Unipro UGENE) with relevant protein domains and sites of posttranslational modifications indicated. **(B)** Percentage of mutual IFITM protein identity (blue) and similarity (yellow). **(C)** One of the proposed models of IFITM protein topology in the plasma/vesicular membrane. IMD – intramembrane domain (amphipathic helix); CIL – conserved intracellular loop; TMD – transmembrane domain. Created with BioRender.com.

A typical feature of these genes is an interferon-stimulated response element (ISRE) located in the 5' flanking promoter/enhancer region (35). The sequence of this element determines the predominant responsiveness to certain types of IFNs. In *IFITM* genes, the ISRE confers responsiveness to both type I (α, β) and II (γ) IFNs (3, 28). In addition to the ISRE, an IFN gamma-activated site (GAS) is also present in the promoters of the *IFITM1*, *IFITM2* and *IFITM3* genes. IFN-insensitive *IFITM5* and *IFITM10* lack both of these elements, and their gene sequences are significantly different from those of the other *IFITM* genes, which is also reflected in the protein sequences (**Figure 1A**) (3).

## Protein structure and intracellular localization

*IFITM* genes encode transmembrane proteins that form a subfamily of Dispanins with a common structure including two transmembrane helices (32). IFITMs are type II transmembrane proteins with a cytosolic N-terminus and a C-terminus located extracellularly or in the endosomal lumen (36, 37) (**Figure 1C**). They may also adopt alternative membrane topologies with the N-terminal domain facing outward. Researchers have not clearly determined whether this topology is a complete inversion of the protein or whether both the N- and C-terminal domains face the ER lumen in this orientation. The frequency of this minority topology is also not clear, and notably, it has been observed in only one of two cell lines tested (36).

The membrane topology of IFITM proteins is not fully understood. Previously, researchers assumed that both α-helix domains are transmembrane regions (33, 38). This hypothesis was recently refined: only one of these two domains is a transmembrane region, while the second is an intramembrane region (39, 40). Chesarino et al. (41) studied the topology of IFITM3 and predicted that the intramembrane domain (IMD) is an amphipathic helix responsible for functional activity. The transmembrane domain (TMD) and IMD are linked by a conserved intracellular loop (CIL) (32). IFITM proteins share a highly evolutionarily conserved CD225 domain (named after the alternative IFITM1 nomenclature, which might be confusing) that extends over the IMD domain and CIL, confirming the presence of an important structural and functional core in IFITM proteins (42). In contrast, the N- and C-terminal domains are highly variable among the members of the IFITM family. Sequential differences provide diverse sites targeted by posttranslational modifications (**Figures 1A, B**), which may be a cause of the varying localization and functions of particular IFITM proteins.

IFITM1 is presumed to be predominantly exposed on the cell surface; therefore, it was formerly denoted as Leu-13 antigen and assigned the cluster of differentiation (CD) number CD225 (5, 6). In contrast, IFITM2 and IFITM3 are localized primarily in endosomes and lysosomes (7). Other studies have documented the presence of IFITM1 and IFITM2 in the mitochondria (43, 44).

However, the intracellular localization of IFITM proteins may change depending on the phosphorylation of particular tyrosine residues in the N-terminal domain. IFITM2 and IFITM3 differ from IFITM1 in the presence of extra N-terminal amino acid residues, including Y20 in IFITM3 and Y19 in the IFITM2 sequence. These tyrosine residues are a part of a sorting motif and provide an endocytic signal targeting IFITM2 and IFITM3 into endosomes. In general, endocytic pathways and intracellular protein trafficking are mediated by adaptor protein (AP) complexes that recognize cargo proteins and direct them into transport vesicles (45). Y20 phosphorylation of IFITM3 hinders recognition by AP-2, which is responsible for IFITM3 endocytosis and thus leads to the accumulation of the IFITM3 protein on the cell surface (7, 46–50). According to the conserved sequences, the same mechanism of internalization is assumed to affect the IFITM2 protein. However, the reported results are contradictory (7, 49). Interestingly, the IFITM3 protein expands from inner cellular organelles to the plasma membrane upon B-cell activation (21, 51). IFITM1 possesses the C-terminal motif KRXX, which regulates its intracellular trafficking and ubiquitination by mediating an interaction with AP-3 (52). However, IFITM1 truncation at the C-terminus or AP-3 knockdown results in localization primarily in the plasma membrane (50, 52). Despite the high conservation among IFITM proteins, fine nuances lead to changes in their structure and intracellular localization that exert a strong effect on their functions. For example, these differences protect cells from viral entry on multiple levels and thus interfere with the diverse viral entry mechanisms developed during evolution.

## Regulation of IFITM expression and function

### Mechanisms regulating IFITM expression under physiological and pathological conditions

**Figure 2:**
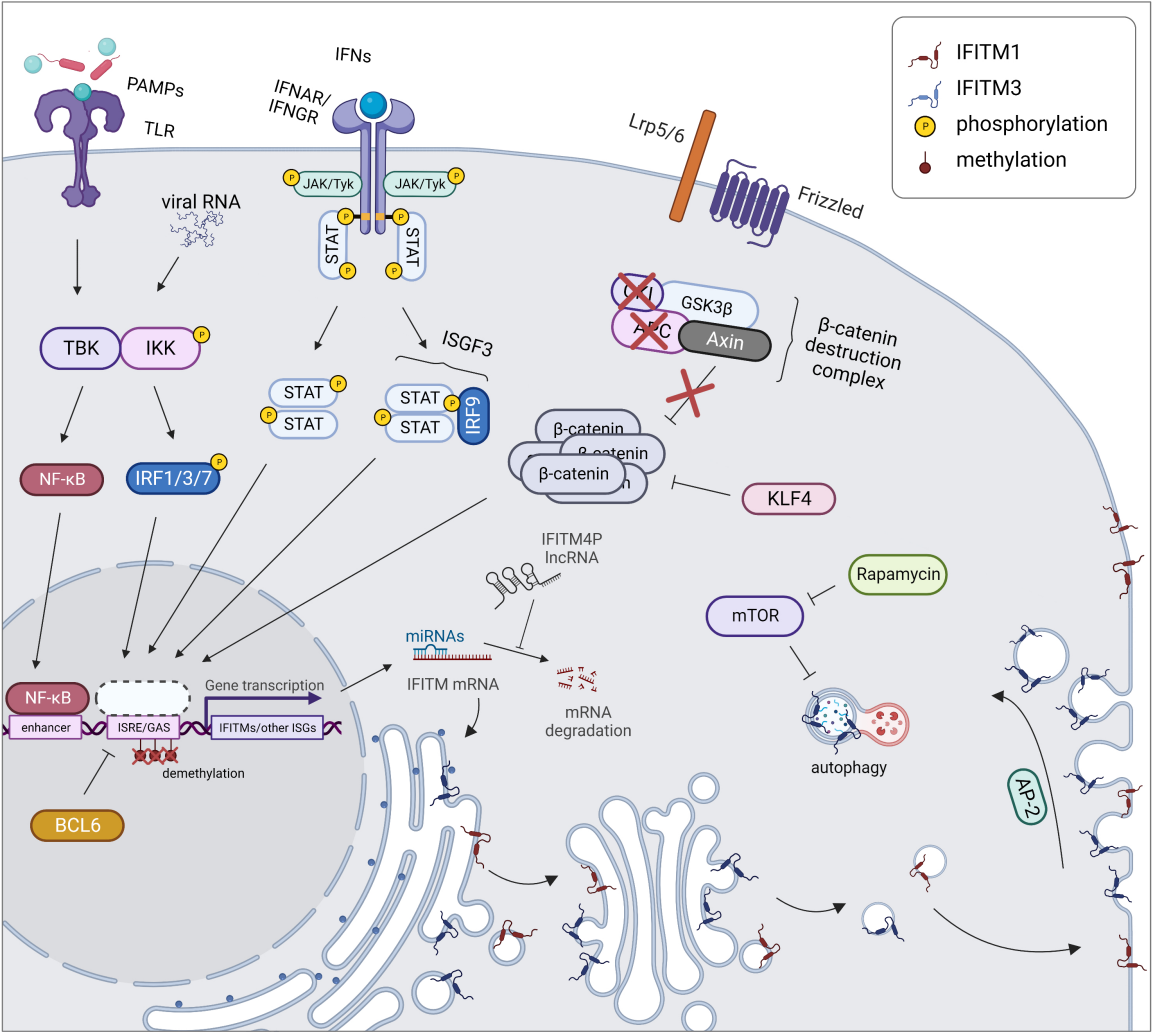
Regulation of expression and localization of IFITM proteins. During an inflammatory response, *IFITM* gene expression is triggered together with other ISGs by STAT/IRF signaling pathways. In cancer cells, the expression of IFITMs is linked with activation of the Wnt/β-catenin signaling pathway which is caused by disruption of β-catenin destruction complex (APC, CKI loss of function). IFITM expression is also controlled by KLF4 inhibiting Wnt/β-catenin signaling. Another cause of increased IFITM level in tumors is promoter demethylation, on the other hand, BCL6 causes transcriptional repression of *IFITM3 *gene by binding to its promoter. Posttranscription regulation of IFITMs includes miRNA-directed cleavage of *IFITM* mRNA, which can be prevented by *IFITM4P* lncRNA acting as a decoy for miRNAs. Posttranslational regulation comprises a proper IFITM localization. While IFITM1 (red) is maintained in the plasma membrane, IFITM3 (blue) is endocytosed into endosomes by AP-2 recognizing its YEML motif. IFITM3 protein turnover is controlled by autophagy. Created with BioRender.com.

During inflammation caused by a pathogen or an autoimmune disease, IFITM genes are regulated in a manner similar to other ISGs (**Figure 2**). The expression and secretion of IFNs by immune (type I and II IFNs) and nonimmune (type I IFNs) cells lead to the stimulation of interferon receptors (IFNAR and IFNGR) in an autocrine and paracrine manner. This stimulation in turn activates Janus kinases (JAKs) and tyrosine-specific kinase (Tyk), which phosphorylate signal transducers and activators of transcription (STATs). This phosphorylation subsequently promotes the translocation of STATs into the nucleus in a homodimeric form or in a complex with IRF9 (together forming ISGF3) and initiation of the transcription of the *IFITM1*, *IFITM2* and *IFITM3* genes along with other ISGs containing ISRE/GAS elements in their promoter region (1, 3, 35). In addition to IRF9, other IRF transcription factor family members (e.g., IRF1, IRF3, and IRF7) induce IFITM expression independent of the STAT pathway (53, 54). Moreover, in addition to IFNs, IFITM expression is stimulated by other inflammatory cytokines (IL-6 and oncostatin M) and angiotensin II, a hormone that increases blood pressure by increasing the intracellular accumulation of calcium (55, 56). Interestingly, IFITM expression may also be induced independently of the IFN-stimulated pathway, secreted cytokines or the typical IFITM inducers IRF3 and IRF7, but the exact mechanism remains unknown (55, 57, 58).

In addition to transient pathogen-induced gene expression, constitutive expression also occurs in some cells. Several cell types, such as stem cells and specific immune cells, express IFITMs constitutively, which provides them with intrinsic immunity against viral infection (18, 59–62).

IFITMs are also constitutively overexpressed in malignant and autoimmune diseases that are associated with an inflammatory response (15, 63–66). Some types of tumors are characterized by chronic activation of the IFN signaling pathway, explaining the upregulation of IFITMs (15, 67). In other tumor types, the exact mechanisms underlying this phenomenon have yet to be described. One of the explanations for the altered regulation of *IFITM* gene expression is the activation of the Wnt/β-catenin signaling pathway, a typical feature of colorectal and ovarian cancers (16, 17, 68, 69). In colorectal cancer cells, this pathway is associated with loss of function of adenomatous polyposis coli (APC), a tumor suppressor protein. Conversely, induction of this tumor suppressor leads to the repression of *IFITM* genes (16). IFITM3 expression is reduced by Krüppel-like factor 4 (KLF4), a known tumor suppressor that interacts with β-catenin and thus inhibits Wnt/β-catenin signaling in the human intestinal epithelium (70, 71).

IFITM2 and IFITM3 are involved in the mammalian target of rapamycin (mTOR) pathway and thus affect the autophagy machinery. mTOR inhibitors decrease the IFITM protein level, not the mRNA level, by enhancing protein degradation in a manner dependent on ubiquitination and endosome acidification. This result reveals a mechanism by which mTOR inhibitors affect lentiviral transduction and also outlines the cause of higher susceptibility of patients to severe acute respiratory syndrome coronavirus 2 (SARS-CoV-2) infection (72, 73). A similar effect is achieved by cyclosporine H through an as yet unexplained mechanism that depends on the presence of phosphorylated Y20 in IFITM3 (74). IFITM3 decreases the IRF3 level through an autophagy-dependent pathway, ensuring a negative feedback loop regulating the IFNβ-stimulated pathway (75, 76). IFITM3 was identified as a factor linking the autophagy protein Ambra1, pSrc and the FAK trafficking network (77).

### Epigenetic regulation of IFITM expression

The expression of *IFITM* genes is also regulated by epigenetic modification of the corresponding promoter. Changes in methylation have been reported after IFN stimulation or in tumors where IFITM overexpression is common. For example, pathological methylation of the *IFITM1* promoter is associated with an elevated level of IFITM1 in gastric cancer (78) and with a metastatic phenotype of ovarian cancer (79). IFITM3 is also regulated epigenetically (80). Demethylation of the *IFITM3* promoter enables binding of the transcription factor Sp1, which increases IFITM3 expression. Hypomethylation is induced physiologically by IFNα stimulation, but it was also detected in human melanoma cells, and the methylation status of the *IFITM3* promoter correlates with hand, foot and mouth disease caused by enterovirus 71 (81). An epigenetic modification that regulates gene expression was also described for *IFITM5*. The transcription factors Sp1 and Sp3 together with Gli2, an executive component of the sonic hedgehog signaling pathway, are responsible for inducing *IFITM5* promoter activity, and this activity is inhibited by CpG methylation of the promoter (82).

Another level of IFITM regulation is achieved by noncoding RNAs that control posttranscriptional mRNA processing. The *IFITM4P* pseudogene, whose transcript serves as a long noncoding RNA, positively affects the expression of the IFITM1, IFITM2 and IFITM3 proteins by competing with the repressive effects of miR-24-3p on the *IFITM1*, *IFITM2* and *IFITM3* mRNA transcripts (83). Several other microRNAs (miRNAs) have been described to regulate *IFITMs*, e.g., miR-36 and miR-29a (84, 85).

### Posttranslational modifications

Sites of posttranslational modifications on IFITMs are highly conserved, indicating their important roles in IFITM functions. Posttranslational modifications of IFITM proteins, such as palmitoylation, ubiquitination, phosphorylation and methylation, determine the intracellular localization, oligomerization and biological functions of these proteins (38, 46, 86). Three potential S-palmitoylated cysteine residues (C71, C72, and C105) were determined by analyzing the IFITM3 sequence with subsequent confirmation of these modifications in IFITM1, IFITM2 and IFITM3 *in vitro* (38). S-palmitoylation affects IFITM antiviral activity by changing the protein conformation, degradation and interaction within the lipid membrane but has no effect on the IFITM subcellular localization (38, 86–88). Interestingly, palmitoylation of the C70 and C71 residues ensures IFITM2 shuttling from hepatocytes to dendritic cells (DCs) by exosomes (89). S-palmitoylation of IFITM5 enables an interaction with FKBP11 in osteoblasts that is important during bone formation (90).

Ubiquitination regulates the presence of IFITM within cellular compartments that influence antiviral activity. The most prevalent ubiquitinated residue in the IFITM3 protein is K24, although ubiquitination also occurs on K83, K88 and K104. Ubiquitin is linked through its K48 and K63 residues to the IFITM3 protein by the ubiquitin ligase NEDD4. K48-linked ubiquitination is associated with protein degradation. A mutation analysis has shown that the lysosomal degradation pathway affects IFITM3 turnover (91). IFITM3 K24-linked ubiquitination permits an interaction with VCP/p97 ATPase, an important element of the ubiquitin system that affects IFITM3 lysosomal trafficking and turnover (92). Interestingly, the interplay between S-palmitoylation and ubiquitination also plays an important role, as these modifications exert opposing effects on the antiviral function of IFITM3 (86).

An important modification of IFITM3 is phosphorylation at the conserved Y20 (IFITM3) residue that is part of the ^20^YXXΦ^23^ endocytic motif. Upon phosphorylation by the tyrosine kinase FYN, the localization of IFITM3 changes from endosomes to the plasma membrane as a result of endocytosis inhibition. Moreover, Y20 phosphorylation reduces IFITM3 ubiquitination, thereby affecting its degradation (46, 49). Although IFITM2 possesses the ^19^YXXΦ^22^ motif, the control of IFITM2 localization is possibly more complex. Narayana et al. (7) confirmed this phenomenon in the Huh-7 hepatocellular carcinoma cell line, but Jia et al. (49) excluded the presence of any phosphotyrosine residues in IFITM2 using HEK293 cells. Interestingly, the localization of the IFITM2 Y19A mutant is only partially changed to the plasma membrane, which might indicate that another mechanism is responsible for IFITM2 internalization (7).

In contrast, methylation does not affect IFITM localization or expression but does modulate antiviral activity. Monomethylation at the K88 residue has been detected in IFITM1, IFITM2 and IFITM3. Further study of the methylation status of IFITM3 revealed that the methyltransferase SET7 was a key modifying enzyme. Viral infection increases IFITM3 methylation by supporting the IFITM3-SET7 interaction and thus impairs IFITM3 antiviral activity. IFNα acts on the IFITM3 methylation status in the opposite manner (93).

### IFITM oligomerization

IFITMs form homo- and hetero-oligomers that result in another level of functional regulation (42, 94). A mutation analysis revealed two phenylalanine residues within the intramembrane domain (F75 and F78) as a key structure ensuring oligomer formation (42). These residues have been shown to be important in the antiviral function of IFITM3 (95). Nevertheless, a subsequent study using a flow cytometry-based fluorescence energy resonance transfer (FRET) approach indicated that these phenylalanine residues are unnecessary for oligomerization (96). Based on the homology between the IFITM3 and PRRT2 proteins, Rahman et al. (10) determined that a conserved ^91^GXXXG^95^ motif is responsible for the oligomeric state, with both glycine residues participating in oligomerization and G95 exerting a stronger effect. The GXXXG motif belongs to the CIL and is typically involved in protein dimerization (10, 97). Surprisingly, mutation of the glycine residues responsible for oligomerization had no effect on the subcellular localization of IFITM3 but significantly suppressed its antiviral properties by preventing membrane rigidification (10). Interesting insights could be provided by studies on IFITM hetero-oligomer formation. The existence of IFITM heterodimers has already been described (42), but a detailed analysis of their functional importance is still lacking. Overall, the growing knowledge of IFITM oligomeric variants suggests a crucial role for oligomerization in the regulation of IFITM functions. However, studies are often focused on a specific IFITM member without relation to the other family members.

## Functions of IFITM proteins

IFITM proteins were first described as adhesive and antiproliferative molecules; however, over time, they have been found to have broad applications in antiviral defense, immune cell functions, bone matrix maturation and germ cell development (51, 98–105). In addition to the most pronounced antiviral properties, the involvement of IFITMs in tumorigenesis, with the most attention given to IFITM1, IFITM2 and IFITM3, is a focus of study. IFITM5 varies substantially from other IFITM protein family members based on its IFN unresponsiveness and lack of known antiviral function (33). A point mutation in the 5' untranslated region (UTR) of the *IFITM5* gene causes osteogenesis imperfecta type V (106). Little is known about IFITM10 to date; nevertheless, it cannot be induced by IFNs and exerts only weak antiviral functions (34). Interestingly, a recent study revealed that IFITM3 is associated with Alzheimer's disease by enhancing γ-secretase activity through a direct interaction, leading to higher production of β-amyloids (107).

## Regulation of membrane structures and antiviral activity

IFITM proteins, with the greatest emphasis on IFITM1, IFITM2 and IFITM3, exert inhibitory effects during viral infection. IFITM10 probably has only a weak antiviral effect (34), and IFITM5 has not yet been connected to such a function; however, little is currently known about these IFITM members. Alber *et* Staeheli (108) were the first to describe the function of IFITM1 as an inhibitor of vesicular stomatitis virus (VSV) and indicated a link between the IFITM protein family and antiviral immunity. The discovery of this cellular innate immune function was not noticed until a study by Brass et al. (109) concerning the antiviral activities of the IFITM1, IFITM2 and IFITM3 proteins against influenza A virus (H1N1), dengue virus and flaviviruses such as West Nile virus was published. Since this rediscovery, the inhibition of many other enveloped and nonenveloped viruses, except for papillomaviruses, adenoviruses, cytomegalovirus, arenaviruses, murine leukemia virus and alphavirus, has been confirmed (109–112). IFITM functions in the context of antiviral immunity have been discussed in great detail elsewhere (8, 113–115).Investigations of the mechanisms by which IFITM proteins act in viral infection help to understand the functions of these proteins within the cell, which may contribute to research in other areas. Based on the almost universal ability of IFITM proteins to block viral infection, this inhibitory activity may not depend on specific receptor recognition. Although the exact mechanism of action is still poorly understood, current knowledge indicates that IFITMs affect the structure and function of membranes in the cell, such as membrane fusion (9, 100), lipid composition within membranes (116, 117), intracellular trafficking of endocytosed cargo (118) and pH regulation in the vesicular environment (119, 120). Additionally, IFITM1 is a known tight junction protein (19, 103).

The role of IFITM proteins seems to be more complicated in regard to coronaviruses. Previously, IFITMs were described to block the entry of SARS-CoV and Middle East respiratory syndrome coronavirus (MERS-CoV) in a manner dependent on the spike protein (121, 122). Recently, extensive research on coronaviruses has revealed a more complex mechanism that depends on many factors, such as the specific cell type, specific members of the IFITM protein family and their intracellular localization, coronavirus species and experimental design. Endogenous IFITMs act as cofactors during SARS-CoV-2 cell entry (123, 124), while ectopic IFITM overexpression blocks infection (94, 123, 125–128). According to Shi et al. (126), IFITM3 acts as a restriction factor for SARS-CoV-2; however, mutation within its endocytosis-promoting YXXФ motif converts IFITM3 into an enhancer of SARS-CoV-2 infection by promoting virus−cell fusion. Although, the virus-inhibiting effect of IFITMs is generally considered nonspecific, affecting mainly the properties of cellular membranes, this may not be the only possibility in the case of coronaviruses. A specific interaction between IFITMs and the spike protein was described recently and IFITMs were even suggested as potential therapeutic targets (123).Syncytium formation is a typical feature of certain viruses from various families, e.g., Reoviridae (129), Retroviridae (130), Paramyxoviridae (131), Herpesviridae (132) and Coronaviridae (128, 133). Virus-infected cells fuse with neighboring cells through surface expression of viral fusion receptors, resulting in a multinucleated cell. This phenomenon helps viruses spread rapidly. IFITMs block virus−cell fusion and syncytium formation induced by all three classes of viral fusogenic proteins divided according to structural criteria (9, 134). Interestingly, this blockade is not mediated through IFITM-mediated interference with specific viral receptors but rather by providing positive curvature of the outer leaflet of the plasma membrane bilayer (9). Buchrieser et al. (128) showed that IFITM1 also blocks cell fusion induced by SARS-CoV-2 and thus precludes syncytium formation, a typical feature of severe coronavirus disease 2019 (COVID-19). IFITM2 and IFITM3 do not exert inhibitory activity, which might be explained by their endosomal localization, excluding them from impacting the cell fusion process dependent on the plasma membrane. Nevertheless, syncytium formation is also a physiological process during syncytiotrophoblast formation. IFITMs also block syncytiotrophoblast formation by inhibiting cell fusion driven by syncytins (endogenous retroviral fusogens). This blockade depends on IFITM palmitoylation, and IFITMs have no effect on syncytin levels. IFN-induced IFITM levels in trophoblasts were shown to affect fetal mortality in mice indicating a possible connection between the excess levels of IFITMs and the pregnancy complications observed during IFN-induced pathologies (105, 135).

IFITMs were described to interact directly with vesicle-associated membrane protein A (VAPA) (116). VAPA is important for lipid homeostasis and membrane trafficking within a cell (136, 137). The IFITM-VAPA interaction causes cholesterol accumulation in late endosomes in the perinuclear area through colocalization with IFITM2 and IFITM3, thereby disrupting cholesterol homeostasis. A probable explanation for this effect is impaired cholesterol endosomal efflux (43), accompanied by changes in endosomal distribution. Cholesterol accumulation itself does not appear to account for virus inhibition (100, 122). Nevertheless, deregulation of cholesterol metabolism has been described in various physiological and pathological processes including tumorigenesis, immune responses and neurodegenerative disorders (138–140). Moreover, IFITMs were described as modulators of the secretory pathway, as their high expression or CIL mutation in the ^81^SVKSRD^86^ motif causes IFITM3 accumulation in the Golgi and subsequently drives trafficking defects (141). Recently, a direct interaction between the amphipathic helix of IFITM3 and cholesterol was reported (117, 142) and F63 and F67 amino acid residues (important for antiviral activity) seem to be responsible for membrane insertion of the amphipathic helix. S-palmitoylation further increases this interaction which might explain the different effects of IFITM proteins on virus cell entry (143). According to Guo et al. (142), the amphipathic helix of IFITM3 is responsible for the negative membrane curvature that is facilitated by cholesterol and other cone-shaped lipids. This statement contradicts Li et al. (9), who described IFITMs as positive curvature promoters, and the negative curvature is predicted to promote membrane fusion (144, 145). A model proposed by Chesarino et al. (41) contests that IFITMs directly affect membrane hemifusion diaphragm formation and suggests that these proteins function as a tape preventing diaphragm expansion. Moreover, the amphipathic helix is well conserved in IFITMs that differ in antiviral and other functions, implying that the resulting effect must be an integration of various properties affecting membranes (posttranslational modifications, protein−protein interactions, and lipid composition).

## Role in tumorigenesis

### Consequences of IFITM overexpression in tumors

**Figure 3:**
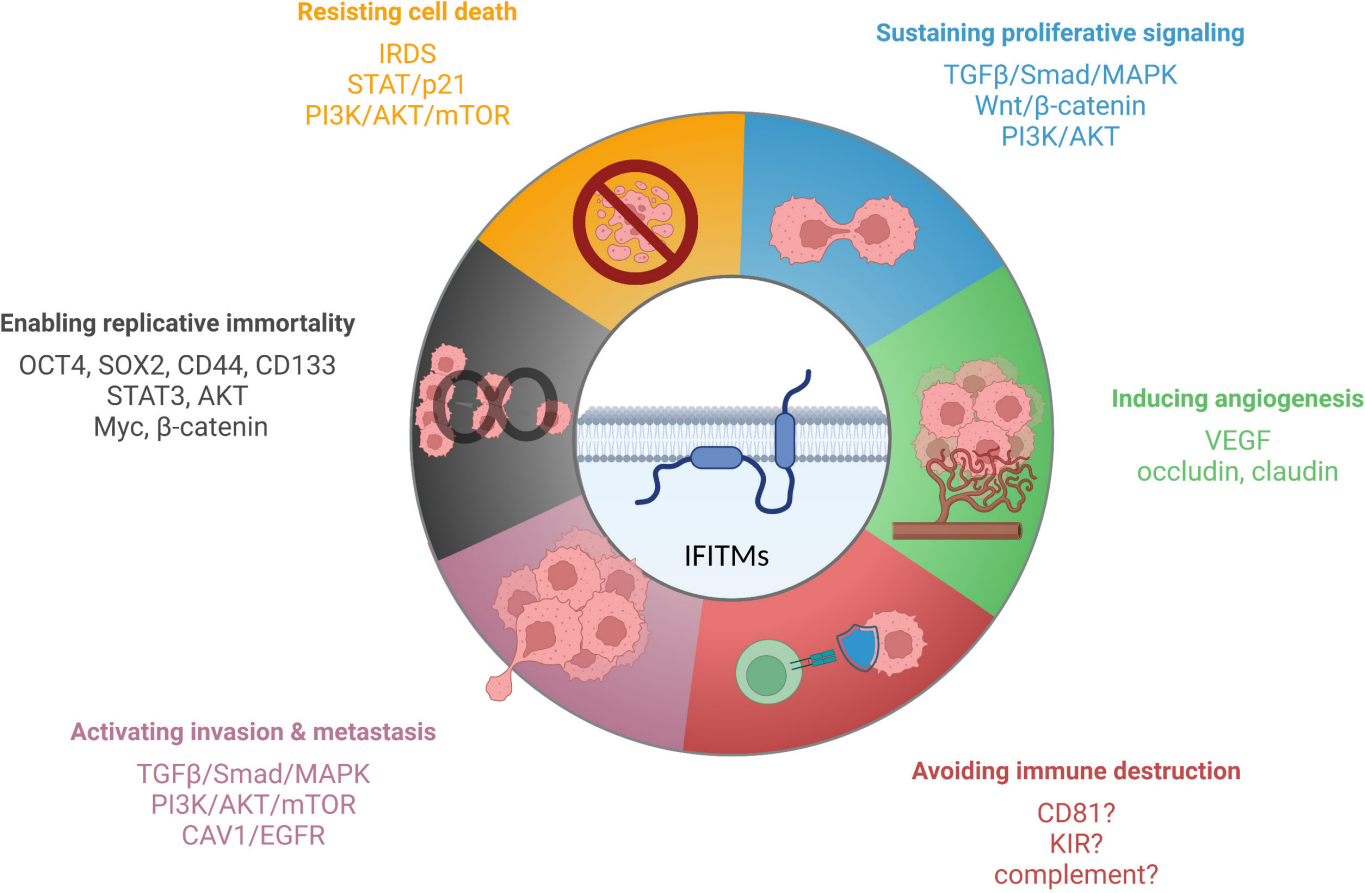
IFITM proteins have been described to affect various cancer hallmarks. The scheme shows IFITM-regulated signaling pathways and factors linked to individual hallmarks of tumor cells. Created with BioRender.com.

IFITM proteins, except IFITM5, which is present primarily in osteoblasts, are expressed in most tissues upon IFN induction (27, 33, 146). Specific cell types (primarily stem cells and certain immune cells) maintain IFITM expression even without IFN stimulation, which endows them with an advantage during viral infection (18, 59–62). Precise regulation of IFITM expression is important because IFITM proteins are functionally involved in tumorigenesis, tumor progression, and relapse (**Figure 3**). However, the molecular basis of IFITM-dependent processes in cancer has not been completely elucidated. Here, we provide a summary of the current knowledge on the roles of the IFITM1, IFITM2 and IFITM3 proteins in different types of tumors and the molecular mechanism, effect on anticancer therapy and links between IFITM-interacting partners with an overlap with tumor progression.

**Figure 4:**
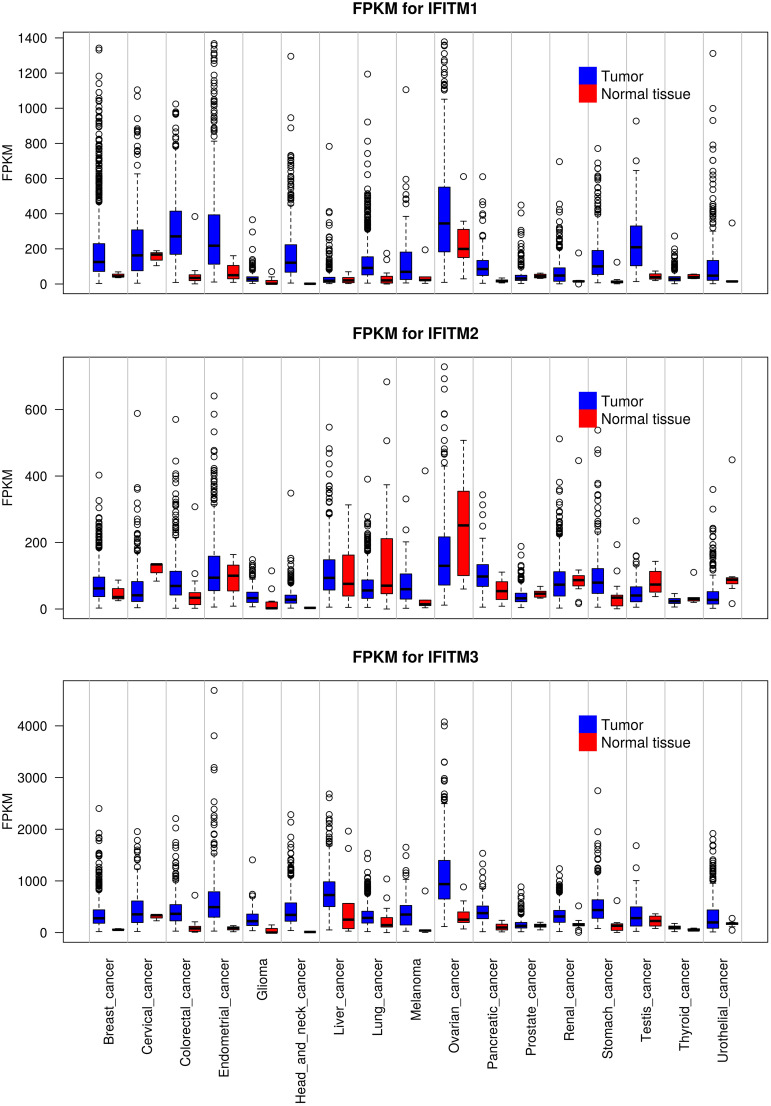
Expression levels of IFITM1, IFITM2 and IFITM3 in different types of tumors compared with adjacent normal tissue. The data used for the analyses were obtained from the GTEx Portal (https://gtexportal.org/home/) on 06.09.2021. The obtained data set was processed using Rscript, which is based on the R language (https://www.R-project.org/), and box plots of the IFITM genes were created. FKPM - fragments per kilobase million.

Deregulated expression of IFITM proteins has been described in most types of solid tumors and hematolymphoid malignancies. To date, IFITM1, IFITM2 and IFITM3 overexpression has been described in various cancer cell lines and tissues. However, differences were observed among the three proteins. IFITM1 and IFITM3 are both overexpressed in most tumor tissues, whereas IFITM2 is overexpressed in only some tumor types and even downregulated in others. Furthermore, in general, IFITM3 is expressed at the highest level, and IFITM2 is expressed at the lowest level in normal and tumor tissues (**Figure 4**, the GTEx Portal). IFITMs are most commonly associated with the pathogenesis of gastrointestinal tract tumors (16, 65, 147–154), and increased IFITM expression has been detected in breast cancer (15, 155–157), prostate cancer (63), lung cancer (17, 158) and hepatocellular carcinoma (64, 159). In addition to carcinomas, which are the most commonly studied tumor types, gliomas (160, 161), acute myeloid leukemia (162) and B-lymphoid malignancies (21) show IFITM3 overexpression. In contrast to IFITM3, IFITM1 and IFITM2 are less frequently assessed in tumor-focused studies.

IFITM expression is associated with the cancer grade and stage. IFITM1 and IFITM3 expression correlates with poorly differentiated gastric, colon and glioma tumor tissues (152, 161, 163, 164). Moreover, IFITM3 expression correlates with lymph node metastasis and distant metastasis in patients with gastric cancer (152). Interestingly, IFITM1 and IFITM3 levels positively correlate with the ER and PR status of breast cancer tissues (157, 165). High IFITM3 expression in patients with acute myeloid leukemia was associated with shorter event-free and overall survival, while no effect of IFITM expression was observed on patients undergoing allogeneic hematopoietic stem cell transplantation (162). Although IFITM2 is less frequently mentioned in tumor-focused studies, this protein seems to act in a similar manner to IFITM1 and IFITM3. IFITM2 upregulation is associated with shorter survival of patients with gastric or renal cancer (166, 167).

The described effects of IFITM proteins on differentiation and invasiveness are consistent with parameters observed during *in vitro* studies of IFITM functions. Increased levels of the IFITM1, IFITM2, and IFITM3 proteins cause higher proliferation of tumor cells, facilitate migration and invasion and influence crosstalk between tumor and immune cells (151, 152, 154, 164, 167).

### Regulation of cancer cell proliferation, cell cycle and cell death

IFITM proteins are closely related to the regulation of cell proliferation. The first studies addressing IFITM functions described IFITM1 as a negative regulator of cell proliferation and a cause of cell cycle arrest in the G1 phase through a mechanism depending on the p53 protein (99, 104, 168). *In vivo* studies of human tumor tissues and tumor-bearing mice focusing on the effects of IFITMs on clinicopathological tumor characteristics found no significant link between IFITM expression and tumor size (64, 157, 158). This result might reflect the fact that the p53 protein is commonly mutated in tumors (169).

On the other hand, *in vitro* studies often describe that targeted IFITM1, IFITM2 and IFITM3 protein silencing in cancer cells causes proliferation inhibition, cell cycle arrest and senescence (15, 17, 150, 154, 157, 161, 167). A study by Daniel-Carmi et al. (149) revealed IFITM2 as a tumor suppressor independent of p53 based on apoptosis induction in colon and lung cancer cell lines. However, recent studies have documented protumorigenic effects of IFITM2 on gastric and renal cancer *in vivo* and *in vitro* (166, 167). IFITM2 thus possibly acts differently in various cancer types, but the cause of this variation is unknown.

In oral squamous carcinoma cell lines (ORL-150 and ORL-204), IFITM3 supports cell proliferation independent of the p53 and p16 pathways, as both cell lines possess deficiencies in these proteins. In contrast, the cyclin D1, CDK4 and pRB proteins are present at reduced levels that explain the observed cell cycle arrest in IFITM3-deficient tumor cells (150). Decreased cyclin D1, β-catenin and c-myc protein levels were detected in the A549 lung cancer cell line after IFITM1 silencing (17).

Despite undisputable IFITM overexpression in tumors, IFITM1, IFITM2, and IFITM3 are known to act as mediators of IFN-induced antiproliferative signals, which remains an unexplained paradox (104, 149, 170). As described for IFITM1, inhibition of cell proliferation is caused by the suppression of the MAPK signaling pathway and stabilization of p53 protein level that results in cell cycle arrest in the G1 phase (104). Surprisingly, IFITM2 inhibits proliferation and subsequently induces apoptosis in a p53-independent manner. However, p53 decreases the IFITM2 protein level (149).

IFITM3 regulates the TGFβ/Smad/MAPK signaling pathway by binding to Smad4, thus activating this pathway (63). IFITM3 also negatively regulates osteopontin (OPN) levels through a direct interaction (171). OPN is a glycoprotein involved in bone mineralization and remodeling, cell attachment, apoptosis regulation and modulation of the immune response (172–174). Moreover, OPN is overexpressed in tumors and correlates with tumor progression and metastasis. In the tumor microenvironment, tumor-derived OPN binds to CD44 on activated T cells, thus apparently suppressing T-cell activation (175). Upon B-cell activation, IFITM3 mediates the amplification signal between CD19 and LYN in proximity to BCR, leading to the activation of PI3K and integrin signaling pathways and PIP3 accumulation in lipid rafts (21). The PI3K and p38 MAPK signaling pathways are activated after the BAG3 protein binds to IFITM2 exposed on the surface of tumor-associated macrophages (176).

IFITM proteins affect cell proliferation according to *in vitro* studies. However, the mechanism underlying this effect remains to be clarified. Some studies describe IFITM proteins as antiproliferative agents, while others have shown the opposite results, which correspond with IFITM upregulation in tumors. Nevertheless, the effect of IFITMs on proliferation is possibly cell type specific and may also depend on the experimental conditions.

### Regulation of cell migration, invasion, and metastasis

The IFITM1, IFITM2 and IFITM3 proteins influence the epithelial–mesenchymal transition (EMT) status, cell migration, invasion and the presence of distant metastases, as has been shown in both *in vitro* and *in vivo* studies performed with various cancer cell lines, animal models and patient samples (15, 16, 64, 71, 151, 154, 155, 164, 166, 167, 177–179). Although IFITM overexpression was described in primary tumor tissues compared to adjacent normal tissues, even higher IFITM1 and IFITM3 expression levels were detected in invasive tumor cells and metastatic lymph nodes draining gastric or colon tumor tissue, indicating a connection between IFITMs and tumor invasiveness and progression (71, 154, 157, 164).

Regarding the molecular mechanism underlying the IFITM-directed modulation of cancer cell migratory abilities, some interesting but still fragmented findings related to several mechanisms and signaling pathways have been reported. The EGFR pathway occupies an important position in tumor development and progression (180). This pathway is negatively regulated by caveolin 1 (CAV1), an interacting partner of the IFITM1 protein (178, 181, 182). IFITM1 acts through the negative regulatory effect of CAV1 on the EGFR pathway and thus affects the EMT signature and migration (164, 183). IFITM1 was defined as a tight junction protein in hepatocytes, in which it interacts with CD81 and occludin to block hepatitis C virus entry. Moreover, IFITM1 promoted the relocalization of CD81 to tight junctions, where it interacted with the proteins occludin and ZO-1 (103). Surprisingly, however, CD81 supports the growth and metastasis of various cancers (184–186) and promotes the IFITM1-induced invasion of head and neck squamous cell carcinoma (HNSCC) cells (187). The effect of IFITM1 on tumor progression is often linked to altered levels of β-catenin, cyclin D1 and c-myc, which might account for the roles of IFITM1 in cancer cell migration, metastatic potential and proliferation (16, 17, 188).

Current knowledge of the molecular mechanism by which IFITM3 confers migration and metastasis benefits to tumor cells shows partial overlap with pathways linked to IFITM1. IFITM3 supports cell migration by activating the PI3K/Akt/mTOR pathway, one of the important pathways in the EMT (159). Further investigations have shown that IFITM1 and IFITM3 affect the p38/MAPK pathway and subsequently stimulate the expression of the extracellular matrix proteases MMP2 and MMP9, which are essential factors contributing to cell migration that act through extracellular matrix remodeling (64, 151, 152, 164, 182, 189). The effect of IFITM3 on MAPK pathway activation is further associated with TGFβ/Smad signaling. IFITM3 is thus a regulator of the TGFβ/Smad/MAPK signaling pathway through the direct interaction between IFITM3 and Smad4, promoting the EMT, cell proliferation, migration and bone metastasis in prostate cancer (63).

IFITM2 and IFITM3 expression is induced after casein kinase 1α (CKIα) ablation in the context of a p53-deficient background. Thus, these proteins are a part of the p53-mediated suppression of the invasiveness signature in enterocytes that accounts for cell polarity and adhesion loss, tissue remodeling and motility (190). CKIα is a part of the β-catenin destruction complex and thus is an essential regulator of the Wnt pathway (191).

### Angiogenesis

Angiogenesis, a process mediating the formation of the vasculature, is essential for achieving proper tissue nutrition under physiological and pathological conditions. The ability of cancer cells to stimulate this process facilitates tumor growth and dissemination. IFITM proteins appear to play an important role in angiogenesis. IFITM1 expression was shown to be upregulated in sprouting endothelial cells, affecting lumen formation. IFITM1-deficient endothelial cells failed to form the lumen properly *in vivo* and *in vitro*. The formation of an intracellular vacuole, the early step in lumenogenesis, was unaffected, while the maturation and expansion stages failed. The observed effects might be explained by the fact that IFITM1 is responsible for the proper localization of the tight junction proteins occludin and claudin-5 (19). Indeed, IFITM1 is associated with an increased microvessel density in lung and breast cancer (165, 192). IFITM1 expression is also stimulated by VEGF, and this effect is dampened by the antiangiogenic drug zoledronate (193). IFITM2 correlates with the expression of VEGF-C, a growth factor of lymphatic vessels. This result suggests that IFITM2 promotes lymph-angiogenesis through VEGF-C signaling and thus facilitates lymphatic metastasis, as shown in renal carcinoma (167).

### Sensitivity to anticancer therapy

IFITM proteins are significantly involved in tumor progression, as summarized above. Moreover, several publications have identified these proteins as molecules affecting the efficacy of anticancer therapies, including radiotherapy, chemotherapy, and endocrine therapy (149, 194–196).

A link among *IFITM1*, *IFITM2* and the cell response to radiation was suggested by Clave et al. (58). The expression of these genes was upregulated after irradiation in the p53-deficient leukemic cell line KG1a, while *IFITM3* expression was not detected even in control samples. Interestingly, IFITM1/2 overexpression was independent of the IFN-stimulated pathway based on the unchanged *IFNγ* and *IRF1* mRNA levels. Later, upregulated IFITM1 was identified together with upregulation of other ISGs in the radioresistant tumor cell line Nu61 compared to the radiosensitive parental cell line SCC-61 (195). Weichselbaum et al. (20) built on this study and identified the IFN-related DNA damage resistance signature (IRDS) as a predictive marker for the efficacy of radio- and chemotherapy among patients with breast cancer. The IRDS contains genes such as *STAT1*, *ISG15*, *OAS-1*, *HLA-A*, *HLA-B* and *IFITM1*. In addition to breast cancer, other human tumor types (head and neck cancer, prostate cancer, lung cancer, and glioma) have been confirmed to exhibit the IRDS using microarray data. Upregulation of an IRDS-like signature containing *IFITM* genes was also identified in gemcitabine-resistant pancreatic cancer cells that were cross-resistant to oncolytic virus therapy (197).

The underlying mechanism of the IRDS might involve the similarity between the effects of viral infection and DNA-damaging agents on tumor cells. During viral infection, the presence of foreign DNA in the cytoplasm of host cells can initiate type I IFN signaling. Erdal et al. (198) reported that DNA-damaging agents used as cancer therapies promoted the release of single-stranded DNA (ssDNA) fragments from the cell nucleus and their accumulation in the cytosol, which led to an IFN-mediated antiviral-like response. The factors that control DNA end resection during double-strand break (DSB) repair were shown to play a major role in generating these DNA fragments.

Yu et al. (199) showed that DSBs induced IFNβ production in an IRF3-dependent manner. IFNβ then acted to amplify DNA damage responses, activate the p53 pathway, promote senescence, and inhibit stem cell function. Therefore, IFN signaling was concluded to serve as a link between DNA damage and apoptosis, cell senescence, stem cell ablation, tissue aging and premature death, which was confirmed using *Terc-* and *Ifnar1-*deficient mice (199). These results support the hypothesis that senescence is primarily focused on eliminating viral infections, as viruses often cause DSBs. IFNβ and ISG induction thus serve as a genome integrity guard.

A possible mechanism underlying the maintenance of the IRDS is the presence of unphosphorylated STAT1 (U-STAT1) in the nucleus, even if the level of the phosphorylated form (YP-STAT1) has returned to the basal level after IFNβ treatment. U-STAT1 maintains a fraction of IFNβ-related immunoregulatory genes, including *IFITM1*, independent of YP-STAT1 (200). U-STAT1 forms the U-ISGF3 transcriptional complex with U-STAT2 and IRF9, which is responsible for maintaining the expression of IRDS genes, ensuring prolonged viral and DNA damage resistance (201, 202). The overexpression of the same subset of genes was also detected in other radioresistant cancer cells and in cancer cells resistant to DNA-damaging agents (203, 204). A recent study identified mucin 1 C-terminal subunit (MUC1-C) as a necessary factor for constitutive IFNβ production and subsequent downstream processes activating the IRDS and DNA damage resistance in triple-negative breast cancer cells (205). In addition to IFITM1, Yang et al. (196) confirmed a connection between IFITM2/IFITM3 and radioresistance based on the GSE expression database. The authors then focused on IFITM1 and verified this result *in vitro* and *in vivo* using oral squamous cell carcinoma cell lines (CAL27 and TSCC-1) and a tumor model established in nude mice. IFITM1 is overexpressed in oral carcinoma tissues, and its expression is even higher after irradiation, at which time it protects cells from death. Interestingly, IFITM1 knockdown was shown to increase the levels of the phospho-STAT1/2/p21 proteins, while the levels of phospho-STAT3/phospho-p21 were decreased. An *in vivo* experiment using a xenograft tumor Balb/c mouse model showed that IFITM1 knockdown in combination with irradiation resulted in distinct tumor suppression compared to irradiation alone. Phosphorylation of p21 at T145 promoted its retention in the cytoplasm (206). Cytoplasmic p21 is a key determinant of cisplatin-based resistance in human testicular carcinoma, as a majority of therapy-resistant patients exhibit a relatively high level of p21 in the cytoplasm (207). High cytoplasmic p21 levels protect carcinoma cells from cisplatin-induced apoptosis (207, 208). Interestingly, cytoplasmic p21 level is inversely correlated with the expression level of Oct4, a stem cell-related transcription factor, and miR-106b, a miRNA linked to the DNA damage response and cancer progression (207, 209, 210). Overexpression of IFITM1 and p27 was detected in a cisplatin-resistant gastric cancer cell line (YCC-3/R) compared to parental YCC-3 cells (211, 212). IFITM1 was also determined to be a critical biomarker of the cisplatin response based on differentially expressed genes identified in a microarray screen of esophageal cancer cell lines (194).

In addition to their connection with DNA-damaging agents, IFITM1 and IFITM3 are positively correlated with the development of aromatase inhibitor resistance in breast cancer cells. This resistance is associated with reduced STAT1 and STAT2 activity, leading to decreased p21 expression through a mechanism independent of p53, a common p21 regulator (165, 213).

In contrast to previous results, IFITM2 knockdown was shown to lead to UV radiation and etoposide (an inhibitor of topoisomerase II) resistance in HeLa cells. This result is consistent with a proapoptotic role for IFITM2 and was found to be p53-independent (149). Existing results indicating similarities in nucleotide/protein sequences among IFITM family members contrast with the functional differences showing that IFITM1 and IFITM3 act in a similar manner but act differently from IFITM2, whose effects on tumor progression and therapeutic efficacy are controversial. This paradox might be a consequence of the use of different tumor models, different posttranslational modifications (e.g., described as missing Y19 phosphorylation), the formation of IFITM homo/heterooligomers or interactions with other proteins. However, quite a few studies have investigated IFITM2 characteristics and functions and compared them with those of IFITM1 and IFITM3; thus, focusing on every single IFITM family member, including its mutual interactions, seems to be necessary.

In summary, IFITM proteins might serve as valuable prognostic and predictive markers for certain solid and hematological tumor types, and as IFITM1 is exposed on the cell surface, it might be exploited as a useful therapeutic target.

### Stem cells

The current knowledge reveals a connection between IFITMs and stem cell maintenance and functions, showing the truly pleiotropic effects of these proteins. Ifitm1, Ifitm2 and Ifitm3 are expressed by primordial germ cells in mice, providing migratory capabilities and affecting the cell distribution during embryonic development (214). However, Ifitms are not essential for proper germline establishment (18, 215). Lipopolysaccharide (LPS) stimulates IFITM1 expression through the binding of the transcription factors NFκB and IRF1 to the R2 enhancer region, and consequently, IFITM1 facilitates the migration of human embryonic stem cells (54).

Even in stem cells, IFITMs work as antiviral proteins. In contrast to terminally differentiated cell types, stem cells are indolent to IFNs but intrinsically express a subset of ISGs, including IFITMs, to help them resist viral infections (18, 216, 217). Interestingly, Wu et al. (18) postulated the differentiation-dependent presence of IFITMs. In stem cells, IFITMs (IFITM1, IFITM2, and IFITM3) are produced constitutively, while this intrinsic production is lost during development into more differentiated cell types.; instead, it is substituted by IFN-dependent expression of IFITMs and other ISGs in the presence of a pathogen.

IFITMs are currently often associated with cancer stemness-related markers. IFITM1 expression was shown to be highly induced after culturing adenocarcinoma cells in stem cell-selective medium (182). Moreover, IFITM1 and IFITM3 confer a sphere-forming ability to various cancers (150, 157, 161, 182). Sphere formation is considered a unique feature of stem cells; therefore, IFITMs might play an important role in cancer stem cell maintenance. Wu et al. (218) described an effect of adipose tissue-derived mesenchymal stem cells on the sensitivity of hepatocellular carcinoma cells to radiotherapy both *in vitro* and *in vivo*. Interestingly, they identified that this effect was influenced by IFITM1 protein expression, as overexpression of this protein caused a loss of these combination therapy benefits.

IFITM1 affects the levels of pAkt, pSTAT3, SOX2, MYC and p-β-catenin, which are linked to cancer stem cell regulation. Moreover, the cancer stem cell markers CD44 and CD133 were shown to be downregulated after IFITM1 depletion. A link between SOX2 and IFITM1 has been indicated, as ectopic SOX2 expression partially restores disrupted processes after IFITM1 depletion, such as the migratory ability and downstream EGFR target gene expression (182).

## Role in immunity

IFITM proteins play important roles in innate immunity, as they fight against infection with a wide range of viruses. Recently, the role of IFITMs in adaptive immunity has also been described. Nevertheless, little and only fragmented information is available about IFITM functions throughout immune processes under physiological and disease conditions. Interestingly, recent studies also suggest a link between IFITM proteins and antitumor immunity. This current knowledge expands the known IFITM functions from antiviral activities through tumorigenic functions to tumor immunosurveillance.

### IFITM expression in immune cells

IFITM proteins have been detected in various human and murine immune cell types, including T cells, B cells, natural killer (NK) cells (219), DCs, monocytes, macrophages (220) and megakaryocytes, which also participate in immunity (221).

The presence of the Leu-13 antigen, corresponding to IFITM1, on the T-cell surface was confirmed by Chen et al. (222). Later, the expression of all three immune-related IFITMs was detected in T cells. Altered Ifitm levels were observed upon murine CD8^+^ and CD4^+^ T-cell activation, but the results have varied among studies. According to Yánez et al. (12), who measured *Ifitm* expression levels in CD4^+^ T cells using RNA sequencing, *Ifitm1* mRNA levels remained low at all observed time points up to 30 hours, and *Ifitm2* was upregulated after T-cell activation, while *Ifitm3* levels rapidly decreased. In contrast, Bedford et al. (57) showed transient elevation of the Ifitm3 protein level upon murine CD8^+^ and CD4^+^ T-cell activation that peaked on the third day after T-cell activation. Surprisingly, this elevated expression was independent of secreted factors, including type I and II IFNs and IL-6, and the transcription factors IRF3 and IRF7, which are known IFITM3 inducers. However, the presence of IFITM3 conferred cellular protection against viral infection, leading to its selectively maintained expression, as was described in lung tissue-resident memory T cells (61).

In B cells, IFITM1 and IFITM3 are detected as a part of a transduction signaling complex. This complex is embedded in the plasma membrane and consists of the surface proteins CD19 and CD21 and tetraspanin protein CD81 (TAPA-1) interacting with IFITM proteins (5, 51, 168, 223, 224). Upon B-cell activation, the IFITM3 level is increased, and the localization of this protein together with that of a complete CD19/CD21/CD81 complex expands from endosomes to lipid rafts on the B-cell surface (21, 51).

IFITM1 and IFITM3 are not present in naïve NK cells. Upon murine cytomegalovirus (MCMV) infection of model mice, the expression of both IFITMs and other early effector factors is transiently elevated in NK cells at day 1.5 after infection (219, 225). Upon influenza infection, IFITM3 is also upregulated in respiratory DCs as a result of IFNγ signaling and the transcription factors IRF3 and IRF7 (226). IFITM proteins also play an important role in megakaryocytes, which regulate IFITM3 expression to activate antiviral immunity, and subsequent IFNα/β secretion also protects bystander hematopoietic stem cells from viral infection (221).

IFITM expression is induced mainly by type I IFNs (particularly IFNβ) in monocyte-derived macrophages (227). In monocytes and macrophages, IFITM transcription is activated by several proinflammatory cytokines (IL-1β, IL-6, and TNF), agonists stimulating TLR signaling pathways and *Mycobacterium tuberculosis* infection. IFITMs are important factors that reduce the growth of these intracellular bacteria in monocytes (55, 220).

### Activation and differentiation of immune cells

Although IFITM proteins are often considered induced after infection, protecting cells from pathogen-induced cell death, IFITMs also mediate the regulation of immune cell functions. The function of IFITM1 and its association with coreceptors of T cells are not clear. A Leu-13-specific monoclonal antibody was shown to cause T-cell clumping, inhibit mitogenic effects induced by CD3 stimulation and fail to stimulate NK-cell activity (222). In contrast to the CD3-stimulated inhibition of proliferation, the Leu-13-specific antibody was found to synergize with CD2-induced proliferation. CD2-stimulated activation is antigen nonspecific and requires cell–cell interactions (228). However, the significance of these studies based on the use of Leu 13-specific antibodies remains to be determined. A T-cell analysis performed with an IFITM-deficient C57BL/6 mouse strain revealed that IFITM proteins regulated T helper (Th)-cell differentiation based on a negative effect on Th1 polarization. However, IFITM proteins might be involved in protecting against allergic diseases. These results are consistent with the evolutionary balance, as a strong cellular immune response leads to susceptibility to allergies. IFITM deficiency in mice also leads to a high susceptibility to colitis-associated tumorigenesis, a deregulated Th17 inflammatory response and increased infiltration of effector CD4^+^ and CD8^+^ T cells and macrophages in colon tissue (229). In these cases, IFITM proteins prevent inflammation and associated tumorigenesis in mice. These studies show that IFITMs are important for maintaining balance during T-cell development and polarizing the T-cell response.

Aggregate formation and the antiproliferative effect of IFITM1 stimulation have also been observed in B cells (230). IFITMs are part of the B-cell coreceptor CD19/CD21/CD81 complex that facilitates antigen-specific B-cell activation and decreases the expression of L-selectin on the cell surface (231). CD81 is involved in signal transduction and cell adhesion in the immune system (223). The interaction of murine Ifitm3 with the tetraspanin CD9 and CD81 proteins was described through immunoprecipitation of splenocyte lysates. No obvious interaction was observed with CD21 or CD19 (51). CD81 also functions as a coreceptor on the T-cell surface. IFITM3 depletion in T cells causes a decrease in the CD3 surface level and dampens TCR signaling (21). Thus, a direct interaction between CD81 and IFITMs might extend their functions from antiviral to antitumoral activities.

IFITM3 has been thoroughly studied in B cells and associated malignancies, as it is associated with a poor clinical outcome. IFITM3 plays substantial roles in B-cell activation and affinity maturation in germinal centers through amplification of the PI3K signaling pathway, which is crucial for the expansion of B cells with high affinity for their cognate antigen. Thus, IFITM3 deficiency impacts antibody production (21, 101). Moreover, relatively low numbers of plasma cells and follicular helper T cells (Tfh cells) were determined to be the result of the upregulation of B-cell lymphoma 6 (BCL6), an inhibitor of plasma-cell and Tfh-cell differentiation (101). Accordingly, Amet et al. (232) described BCL6 as a repressor of antiviral resistance in Tfh cells. BCL6 functions as a transcriptional repressor, binding to the *IFITM3* and *MX2* promoters and thus diminishing their expression. IFITM3 supports transformation processes induced by the oncogenes BCR-ABL1 and NRAS^G12D^. IFITM3 deficiency impairs signaling pathways downstream of BCR engagement through the partial loss of CD19 surface expression and loss of an interaction with LYN, a Src-family tyrosine kinase (21). IFITM3 expression in B-cell progenitors and Ph^+^ acute lymphoblastic leukemia (ALL) cells stabilizes CD19 membrane expression, which affects cell proliferation through positive IL7R regulation and limits the activity of BCR-ABL1 (13). In turn, inhibition of the kinase BCR-ABL1 leads to a decrease in the IFITM3 expression level. The kinases BCR-ABL1 and LYN phosphorylate IFITM3 to promote a change in its localization from endosomes to the plasma membrane, where it is involved in BCR signaling and associated malignant transformation (21).

As innate immune cells, NK cells contribute to immunosurveillance and are essential in the fight against viruses. IFITM3-deficient C57BL/6 mice were found to exhibit enhanced NK-cell activity and function during influenza A virus infection, while the activity of other lung-infiltrated immune cells (lymphocytes, macrophages, and DCs) remained unaffected (233). Infusini et al. (226) reported that IFITM3 helped DCs traffic into lung-draining lymph nodes and thereby ensured the activation of influenza-specific CD8^+^ T cells. NKcell deregulation might cause increased mortality, as abnormal NKcell activation leads to lung tissue injury. These results suggest an important link between IFITM proteins and the regulation of immune cells, which might be affected during both antiviral and antitumor immune processes. As IFITM1 and IFITM3 have been described as factors regulating the proliferation of lymphocytes, the same function seems to be mediated in active NK cells (168, 230). The precise regulation of immune cell counts, and subsequent functions is important for efficient immune responses and prevention of autoimmune/allergic states.

### Modulation of cytokine production

In addition to blocking virion entry through the plasma or endocytic vesicle membrane, IFITMs restrict viruses by modulating the production of cytokines, which in turn act on various immune cells. In a murine herpesvirus model (MCMV), IFITM3 did not block virion cell entry or viral replication but limited the production of cytokines, especially IL-6, thus preventing T and NKcell death (234). Subsequent research confirmed this phenomenon in a human herpesvirus model (HCMV), and moreover, the authors elucidated the mechanism. They suggested that IFITM3 drives Nogo-B turnover through a direct interaction, which in turn affects TLR signaling pathways and finally proinflammatory cytokine production (235). A murine influenza model revealed that IFITM3 dampened the cytokine storm typical of respiratory viruses based on the observation of increased inflammatory and apoptotic responses together with pathologically activated NK cells in the lungs and spleen of IFITM3-deficient mice (233). This effect was also observed in patients infected with H7N9 influenza carrying the IFITM3 rs12252-C/C genotype (single-nucleotide polymorphism (SNP) affecting antiviral functions). In this case, the patients exhibited high plasma cytokine (particularly IL-6, IL-8, and MIP-1β) levels that were associated with a poor clinical outcome (14).

In IFITM3-deficient mice with induced colitis, an inflammatory disease of the intestines, relatively high production of proinflammatory cytokines (IL-17, TGFβ, TNFα, and IL-6) was observed. This cytokine imbalance aggravated symptoms of colitis and potentiated the development of a Th17 response (229). An effect of IFITMs on murine CD4^+^ Th-cell differentiation was described in an asthma model, in which IFITM depletion caused enhanced development of a Th1 response. CD4^+^ cells from IFITM-deficient mice expressed lower levels of IL-4 and IL-13 (cytokines typical of Th2 development), while the levels of Th1-supporting cytokines, such as IFNγ and IL-27, were higher (12).

### Inflammation and tumorigenesis

During tumor development occurring in the context of immune surveillance, the phenomenon of immune escape may occur. Initially, this process comprises tumor elimination by innate immunity (NK cells), but inconsistent elimination functions as a tool for selecting transformed cells. Later, adaptive immunity (e.g., T cells) is activated to eliminate damaged cells. Surviving transformed cells can coexist with immune cells in an equilibrium state, but ultimately, selected individuals escape immunosurveillance, enabling them to undergo subsequent progression. Many immune evasion mechanisms have been identified in tumors. Among those that are frequently described are the downregulation of MHC complexes and altered levels of other proteins (e.g., PD-L1 and B7-H4) on the tumor cell surface. Increasing attention has been directed to the role of IFNγ in the antitumor immune response. Although the results are controversial, the concentration and exposure time of IFNγ in the tumor microenvironment seem to be crucial (236, 237). Moreover, the IFNγ signature defined by IFNγ-related gene levels in tumors may help predict the patient response to immunotherapy.

IFITM proteins, members of the ISG group, are indisputably linked to antiviral immunity and tumor progression, but their role in antitumor immunity is not clear. Here, we summarize the current knowledge of IFITM proteins, the related regulation of immunity and their effects on tumor surveillance. A pancancer analysis of transcriptomic data showed positive correlations between IFITM3 expression and immunomodulators (HLA, chemokines, and immunostimulators), tumor-infiltrating immune cells and immune checkpoints. The correlation between IFITM3 expression and the inflammatory status in the tumor microenvironment might be beneficial for the identification of "hot" tumors (238). Shen et al. (239) determined the global transcriptional program after IFITM3 knockdown in the HeLa cell line to identify differentially expressed genes. They identified 1011 downregulated and 615 upregulated transcripts in various pathways, such as complement and antigen processing and presentation. The majority of components in the complement pathway were downregulated after IFITM3 knockdown. Although the complement system contributes to immune surveillance, increased expression of its components was detected in malignant tumors, confirming that these components may participate in immunosuppression (240). Antigen processing is essential for T-cell activation. MHCII downregulation has been described after IFITM3 depletion. The link between MHC complexes and IFITM proteins has been further documented by Gómez-Herranz et al. (241). They described a protein–protein interaction between HLA-B and the IFITM1/3 proteins in the SiHa cervical cancer cell line, and IFITM1/3 knockout was shown to lead to HLA-B downregulation. Interestingly, formalin-fixed, paraffin-embedded (FFPE) samples of IFITM1/3-negative cervical cancer were associated with the metastatic potential. Moreover, IFITM1 silencing led to decreases in HLA class I, IFITM3 and ISG15 expression. The association of IFITM1 with immunogenicity was also suggested in a pancreatic cancer model, in which upregulated IFITM1 expression levels had protumorigenic functions; however, IFITM1 was positively correlated with the number of tumor-infiltrating immune cells (242). A study focused on kidney cancer confirmed positive correlations between immune cell infiltration and the expression of all three immune-related IFITMs (243).

In contrast, IFITM3 expression in HNSCC samples was positively correlated with immunosuppressive proteins such as programmed cell death ligand 1 (PD-L1), interferon induced protein with tetratricopeptide repeats 1 (IFIT1), B7-H7, V-domain Ig suppressor of T-cell activation (VISTA), indoleamine 2,3-dioxygenase (IDO), CD68, CD163 and CD206 (244). Further supporting the link between IFITM and immune suppression, IFITM1 levels were found to be elevated in myeloid-derived suppressor cells (MDSCs) compared with myeloid counterparts, namely, monocytes and neutrophils, isolated from the mouse mammary tumor virus-polyomavirus middle T antigen (MMTV-PyMT) transgenic model of breast cancer (245). Increased numbers of infiltrated effector T cells and macrophages were also detected in the colon tissue of IFITM-deficient mice, leading to higher susceptibility to colitis-associated tumorigenesis (229).

Yang et al. (154) confirmed that IFITM1 renders gastric tumor cells resistant to NK cells. This effect was shown to be independent of other cell-surface molecules, such as HLA class I (negative NK-cell regulator), ULBP1 (NK-cell activator), CD54, CD11a and Fas. Contacts between tumor and NK cells remained unaffected; thus, IFITM1 does not function as an interfering agent. Further analysis of NKcell signaling and changes in the proteome should be analyzed, as IFITM proteins are associated with various proteins modulating NKcell activity, such as CD81 and KIR receptors (51, 239). The positive correlation and interaction between IFITM1 and CD81 potentially expands the effect of IFITM1 on antitumor immunity, as CD81 plays an important role in the activities of tumor-infiltrating immunosuppressive cells (MDSCs and regulatory T cells (Tregs)) (185, 187).

IFITM2 and IFITM3 are shuttled between cells by exosomes, and transferred IFITM3 exhibits expanded antiviral and antibacterial effects (246, 247). IFITM2 serves as a negative regulator of anti-hepatitis B virus (HBV) immunity, as IFITM2 overexpressed in hepatocytes is released in exosomes that produce a subsequent damping effect on IFNα production by DCs. This mechanism also neutralizes the beneficial effect of exogenous IFNα therapy on patients with chronic hepatitis B (89). Given the similar protein sequences among IFITM members, IFITM1 is possibly transferred by this mechanism, but no evidence exists to date. Only *IFITM1* mRNA transfer has been described between macrophages and hepatoblastoma cells (248). IFITM1 expression reduces exosome uptake by colorectal cancer cells, while exosome release is not affected (249). In the cancer context, an investigation of IFITM-directed vesicle transfer between tumor and immune cells might contribute to understanding the mechanism by which IFITMs affect antitumor immunity.

In conclusion, IFITM proteins have irreplaceable roles in immune cell development, activation, and antiviral defense. Even if IFITMs are associated with tumor immunogenicity through regulation of MHC complex exposure on the tumor cell surface, they also correlate with immunosuppressive factors that allow tumor cells to escape immune surveillance. Further investigations are needed in this field to reveal the more complex landscape of IFITM functions in the tumor microenvironment.

## Summary and future prospects

IFITM proteins form an evolutionarily conserved protein family. Despite the highly similar protein core indicating an important role for ensuring physiological functions, minor changes in the sequence structure among IFITM proteins potentially lead to significant differences in function. Because of the high similarity, an examination of each IFITM protein separately has been difficult in the past. However, the recent development of specific antibodies has alreadyfacilitated these experiments, leading to an increasing number of studies describing the difference between IFITMs. Posttranslational modifications, oligomerization and interactions with other proteins make the investigation even more complex. This diversity has been shown to be beneficial to ensure a broad range of functions. IFITM proteins are widely studied as antiviral factors. However, poor regulation and a high expression have been described in many cancer types, linking IFITMs to tumor cell proliferation, sensitivity to therapy and cancer progression. Recent studies have shown the involvement of IFITMs in both innate and acquired immunity, as they regulate immune cell functions and development. The resulting function of IFITMs thus depends strongly on spatiotemporal and cell-specific IFITM expression. Current research describing the association of IFITM proteins with antitumor immunity opens another interesting field of study of these proteins, which might provide new insights into tumor immunotherapy.

## Author contributions

NF wrote the first draft of the manuscript, FZK analyzed the data, and MN wrote sections of the manuscript and performed a major revision. TH, BV performed a final proofreading step with comments. All authors contributed to the article and approved the submitted version.

## Funding

This work was funded by the European Regional Development Fund (Project ENOCH, CZ.02.1.01/0.0/0.0/16_019/0000868), the Ministry of Health, Czech Republic (Conceptual Development of Research Organization MMCI, 00209805) and the Czech Science Foundation (GACR 22-02940S).

## Conflict of interest

The authors declare that the research was conducted in the absence of any commercial or financial relationships that could be construed as a potential conflict of interest.

## Publisher’s note

All claims expressed in this article are solely those of the authors and do not necessarily represent those of their affiliated organizations, or those of the publisher, the editors and the reviewers. Any product that may be evaluated in this article, or claim that may be made by its manufacturer, is not guaranteed or endorsed by the publisher.

## Glossary

**Table:**

IFN	interferon
IFITM	interferon-induced transmembrane protein
ISG	interferon-stimulated gene
ISRE	interferon-stimulated response element
GAS	IFN gamma-activated site
IMD	intramembrane domain
TMD	transmembrane domain
CIL	conserved intracellular loop
CD	cluster of differentiation
AP	adaptor protein
IFNAR	interferon α/β receptor
IFNGR	interferon γ receptor
JAK	Janus kinase
Tyk	tyrosine specific kinase
STAT	signal transducer and activator of transcription
IRF	interferon regulatory factor
APC	adenomatous polyposis coli
KLF4	Krüppel-like factor 4
mTOR	mammalian target of rapamycin
SARS-CoV	severe acute respiratory syndrome coronavirus
DCs	dendritic cells
VSV	vesicular stomatitis virus
MERS-CoV	Middle East respiratory syndrome coronavirus
VAPA	vesicle-associated membrane protein A
FKPM	fragments per kilobase million
OPN	osteopontin
CAV1	caveolin 1
CKIα	casein kinase 1α
IRDS	IFN-related DNA damage resistance signature
DSB	double-strand break
MUC1-C	mucin 1 C-terminal subunit
Tfh cells	follicular helper T cells
BCL6	B-cell lymphoma 6
MCMV	murine cytomegalovirus
PD-L1	programmed cell death ligand 1
IFIT1	interferon induced protein with tetratricopeptide repeats 1
VISTA	V-domain Ig suppressor of T-cell activation
IDO	indoleamine 2,3-dioxygenase
MDSC	myeloid-derived suppressor cell
MMTV-PyMT	mouse mammary tumor virus-polyomavirus middle T antigen
